# High levels of AAV vector integration into CRISPR-induced DNA breaks

**DOI:** 10.1038/s41467-019-12449-2

**Published:** 2019-09-30

**Authors:** Killian S. Hanlon, Benjamin P. Kleinstiver, Sara P. Garcia, Mikołaj P. Zaborowski, Adrienn Volak, Stefan E. Spirig, Alissa Muller, Alexander A. Sousa, Shengdar Q. Tsai, Niclas E. Bengtsson, Camilla Lööv, Martin Ingelsson, Jeffrey S. Chamberlain, David P. Corey, Martin J. Aryee, J. Keith Joung, Xandra O. Breakefield, Casey A. Maguire, Bence György

**Affiliations:** 1 Department of Neurobiology, Harvard Medical School, Boston, MA 02115 USA; 2 Molecular Neurogenetics Unit, Department of Neurology, Massachusetts General Hospital, Charlestown, MA 02129 USA; 3 Center for Genomic Medicine, Massachusetts General Hospital, Boston, MA USA; 4 Department of Pathology, Massachusetts General Hospital, Boston, MA USA; 5 Department of Pathology, Harvard Medical School, Boston, MA USA; 6 Molecular Pathology Unit, Massachusetts General Hospital, Charlestown, MA 02129 USA; 7 Center for Cancer Research and Center for Computational and Integrative Biology, Massachusetts General Hospital, Charlestown, MA USA; 8 Program in Neuroscience, Harvard Medical School, Boston, MA 02115 USA; 9 Department of Gynecology, Obstetrics and Gynecologic Oncology, Division of Gynecologic Oncology, Poznań University of Medical Sciences, 60-535 Poznań, Poland; 10 Institute of Molecular and Clinical Ophthalmology Basel, 4031 Basel, Switzerland; 11 Department of Hematology, St. Jude Children’s Research Hospital, Memphis, TN USA; 12 Department of Neurology, University of Washington, Seattle, WA 98195 USA; 13 Uppsala University, Department of Public Health and Caring Sciences, Geriatrics, Uppsala, Sweden; 14 Department of Biostatistics, Harvard T. H. Chan School of Public Health, Boston, MA USA

**Keywords:** Targeted gene repair, CRISPR-Cas9 genome editing, Neuroscience

## Abstract

Adeno-associated virus (AAV) vectors have shown promising results in preclinical models, but the genomic consequences of transduction with AAV vectors encoding CRISPR-Cas nucleases is still being examined. In this study, we observe high levels of AAV integration (up to 47%) into Cas9-induced double-strand breaks (DSBs) in therapeutically relevant genes in cultured murine neurons, mouse brain, muscle and cochlea. Genome-wide AAV mapping in mouse brain shows no overall increase of AAV integration except at the CRISPR/Cas9 target site. To allow detailed characterization of integration events we engineer a miniature AAV encoding a 465 bp lambda bacteriophage DNA (AAV-λ465), enabling sequencing of the entire integrated vector genome. The integration profile of AAV-465λ in cultured cells display both full-length and fragmented AAV genomes at Cas9 on-target sites. Our data indicate that AAV integration should be recognized as a common outcome for applications that utilize AAV for genome editing.

## Abstract (web summary)

In-depth characterization of adeno-associated virus (AAV)-mediated CRISPR delivery is still lacking. Here, the authors show high levels of integration into Cas9-induced double-strand breaks (DSBs) in therapeutically relevant genes in vivo.

## Introduction

Genome editing with RNA-guided nucleases holds great promise for the treatment of human diseases. Recently, there have been several advances in addressing some of the key challenges in the case of in vivo genome editing, such as increasing specificity^1–3^ and developing delivery tools for in vivo applications^4,5^. In particular, delivery of CRISPR/Cas9 nucleases by AAV vectors has shown therapeutic benefit in multiple preclinical models of diseases^6–10^ and this modality is moving quickly towards clinical trials^11^. AAV offers safe and stable transgene expression levels in vivo in many differentiated tissues, and the recent FDA approval of the AAV-based gene therapy product *Luxturna* places this vector at the forefront of gene therapy. However, long-term expression of certain transgenes (e.g., Cas9) from an AAV vector might lead to genotoxic effects due to the sustained activity of an active nuclease. Currently, there is limited in-depth characterization of potential outcomes of AAV-mediated CRISPR delivery, particularly in target tissues after in vivo administration. It is well known that the majority of AAV vectors exist in an extrachromosomal state, however, it has been shown previously that a fraction of AAV vectors integrate into pre-existing double-stranded breaks (DSB)^12,13^. Furthermore, non-homologous end-joining (NHEJ)-mediated integration of AAV vectors was also observed after DSBs induced by zinc-finger nuclease or CRISPR in liver^14^, and in muscle^15^, and eye^11^, respectively. There has been limited in-depth characterization of AAV integration into nuclease-induced breaks in non-dividing cells in vivo.

In this study, we analyze integration of AAV vectors genome-wide and into CRISPR-induced DSBs in vivo focusing on therapeutically relevant target genes in differentiated cells of the nervous system (*APP*^*SW*^, *Mecp2*, *Dnmt3b*), muscle (*Dmd*) and the inner ear (*Tmc1*). *APP* and human *TMC1* are carriers of important dominantly inherited mutations that cause early-onset Alzheimer’s disease and progressive deafness^16^, respectively, whereas *MECP2* is a potential target gene in Rett syndrome^17^. *DNMT3B* is expressed during neurodevelopment and is involved in DNA methylation^18^. Gene editing is a very promising strategy for Duchenne muscular dystrophy, as exon deletion or homologous recombination after CRISPR targeting can restore dystrophin expression with improvement in muscle function^6,15,19–21^. Surprisingly, we observe high integration frequencies of AAV sequences at the CRISPR cut sites of all on-target genes. Using miniature AAV vectors we demonstrate that integrated vector genomes can be fragmented, full length, or be present as concatemers. In vivo genome-wide mapping of AAV vectors in the brain shows integration into multiple sites, including the CRISPR on-target site. However, outside of the CRISPR target region, genome-wide AAV integration rates are not different between an AAV control vector and AAV carrying Cas9 and gRNA, suggesting that Cas9 does not lead to widespread genotoxic effects in the brain.

## Results

### AAV vector sequences are detected at CRISPR-induced DSBs

**Fig. 1:**
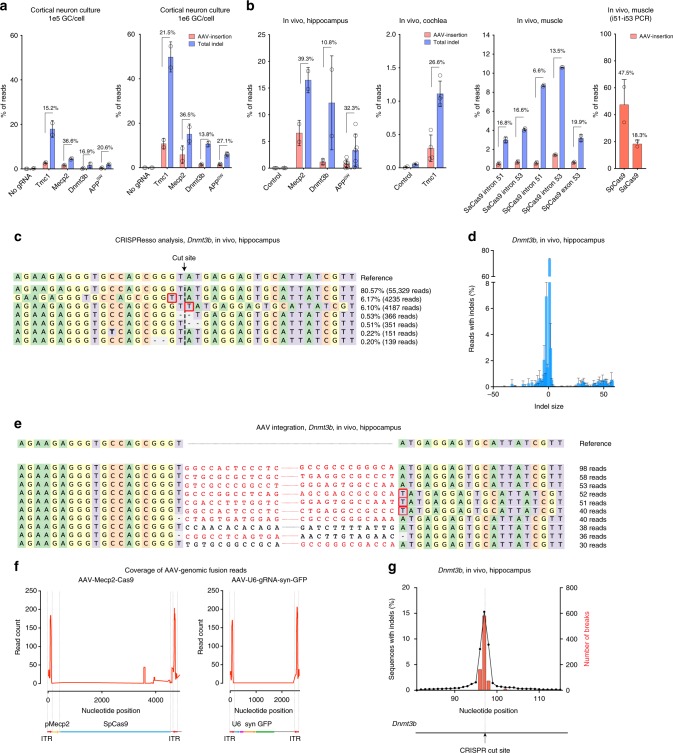
AAV vectors integrate into CRISPR/Cas9 cut sites in vitro and in vivo. **a** Primary murine cortical neurons were transduced with an AAV1 vector encoding Cas9 as well as another AAV1 vector encoding a guide RNA (gRNA) against the genes indicated. Two different doses, 1e5 gc/cell (left panel) and 1e6 gc/cell (right panel), were tested. The negative control was neurons transduced by AAV-Cas9 vector without gRNA (*Tmc1* gene was amplified by PCR for 1e5 gc/cell and *APP* gene was amplified for 1e6 gc/cell). Frequency of AAV sequences present at indels at the target site are shown in red vs total number of indels in blue. AAV capture efficiencies are shown as percentages on the graphs. Two biological replicates were sequenced for each condition (3 for *APP*^*SW*^ gene, 1e6 gc/cell dose). **b** AAV integration into Cas9 cut sites targeting therapeutic genes in the murine hippocampus, cochlea or muscle. For *APP*^*SW*^, non-injected cerebellum or cortex was used as control. For *Tmc1*, non-injected cochleas were used. Animal numbers and the number of sequencing reactions are as follows (numbers of animals pooled per reaction included in parentheses): Hippocampus, control: *n* = 3 (3 reactions), *Mecp2*: *n* = 5 (2 reactions, *n* = 3 and 2), *Dnmt3b*: *n* = 5 (2 reactions, *n* = 3 and 2), *APP*^*SW*^: *n* = 7 (7 reactions). Cochlea samples, non-injected: *n* = 21 (2 reactions, *n* = 9 and 12), injected: *n* = 33 (4 reactions, n = 6, 6, 9, and 12 animals). Muscle samples: *n* = 8 (2 reactions, *n* = 4 and 4). **c** CRISPResso analysis showing small indels at cut site from hippocampus, injected with AAV-Cas9 and AAV-gRNA against *Dnmt3b*. **d** Bimodal distribution of indel sizes, the larger indicating AAV sequence integration at the cut site, with specific examples shown in **e** (two sequencing reactions from 2 and 3 animals, respectively). **f** Characterization of AAV vector region present in indels with AAV-Mecp2-Cas9 (left panel) and AAV-U6-gRNA-syn-GFP (right panel) in brain samples (*Dnmt3b* was targeted). **g** Distribution of AAV integration surrounding the CRISPR cut site in the case of hippocampus, *Dnmt3b* was targeted (two sequencing reactions from 2 and 3 animals, respectively). Bars represent mean ± SD. Source data are provided as a Source Data file

In analyzing next-generation sequencing (NGS) data from an in vivo CRISPR gene therapy approach targeting the *Tmc1*^*Beethoven*^ mutation in the inner ear, we observed AAV inverted terminal repeat (ITR) sequences within CRISPR indels^22^. To confirm this observation and expand these findings to other genes, we first analyzed AAV vector integration in cultured cortical neurons derived from wild type (WT) C57BL/6 mice. Cells were treated with AAV1 carrying *S. pyogenes* Cas9 (SpCas9) and separate AAV1 vectors carrying gRNAs against the wild type (WT) coding sequence of *Mecp2*, *Dnmt3b* or *Tmc1*, at 10^5^ or 10^6^ vector genomes (vg) per cell. Cells were incubated with AAV vectors for 1 day and then kept in culture for another 20 days before harvesting genomic DNA to assess AAV integration (Fig. 1a). Genomic DNA was amplified by a high-fidelity polymerase using a very long extension time (see Methods) to allow for inclusion of potentially large integration events. Deep sequencing of PCR products from the region flanking the cut site revealed characteristic indel formation in all targeted genes (Fig. 1a and Supplementary Data 1), with the majority of indels observed being single nucleotide changes. Analysis of the percentage of reads that aligned to AAV sequences revealed that for all target genes, fusion reads between AAV vector genome and host genome were detectable at variable efficiencies (0.06–12.5%; Fig. 1a). To quantify AAV integration efficiency as a fraction of the total nuclease-induced events, reads that contained AAV sequences were normalized against all reads that harbored insertions or deletions compared with the reference sequence (including AAV integration). This measure is termed AAV capture ratio. We found the AAV capture ratio varied between 13.8% and 36.5% among different targets (Fig. 1a) and was not significantly different between the two different vg/cell conditions (paired *t*-test, *p* = 0.224). As expected, the higher dose of AAV-Cas9 and AAV-gRNA led to a higher percentage of reads with indels. For example at 10^5^ gc/cell with the *Tmc1* specific gRNA, there were 18.0% of reads with indels, while this increased to 49.8% at 10^6^ gc/cell. We also reanalyzed genomic DNA from our previous study^8^ using mouse primary cortical neurons, which overexpress a mutated form of human *APP* gene (amyloid precursor protein with the Swedish mutation, *APP*^*SW*^ from the *Tg2576* strain). In this study^8^, the experimental conditions were the same as in the present study. Similarly, we observed AAV integration into *APP*^*SW*^, with capture ratios of 20.6% and 27.1% for 10^5^ or 10^6^ vg/cell, respectively. These results suggest that a substantial number of gene editing outcomes in non-dividing cultured neurons are a result of AAV vector integration at the on-target site.

### Vector integration into therapeutically relevant genes in vivo

Next, we analyzed AAV vector integration into CRISPR-induced breaks in vivo in three different organs (brain, cochlea, and muscle). We first analyzed in vivo AAV integration in the brain by performing intrahippocampal injections of separate AAV1 vectors expressing Cas9 and gRNAs targeting either the *Mecp2* or *Dnmt3b* genes (5 × 10^9^ vg from AAV-Cas9 and 3 × 10^9^ vg from AAV-gRNA). Similar to above, we also reanalyzed genomic DNA isolated from hippocampus tissue from human *APP*^*SW*^ transgenic mice (*T*g2576) treated with AAVs encoding Cas9 and a gRNA targeting the *APP*^*SW*^ gene^8^. We observed indel formation and AAV integration at the CRISPR on-target site for all three target genes in vivo in the hippocampus (Fig. 1b and Supplementary Data 2). AAV capture ratios (reads with AAV integration normalized to reads with indels) were found to be 39.3%, 10.8%, and 32.3% for *Mecp2*, *Dnmt3b*, and *APP*^*SW*^, respectively.

In the cochlea, we analyzed data from our previous study^22^ and found AAV integration into *Tmc1* (Fig. 1b). In this study, an allele-selective *S. aureus* Cas9 (SaCas9-KKH^23^) was used to target the *Beethoven*^16^ mutation, and AAV-mediated allele-selective disruption led to a complete halt of hair cell degeneration and hearing preservation up to 1 year post injection^22^. Integration was found in the *Tmc1* gene in injected animals with a capture ratio of 26.6% (Fig. 1b).

We also analyzed genomic DNA from the study by Bengtsson et al.^6^ to assess AAV integration in muscle tissue in vivo. In this study, mice received intravenous injections of AAVs carrying either SpCas9 or *S. aureus* Cas9 (SaCas9) and gRNAs targeting the *Dmd* gene. We observed AAV integration in all CRISPR target sites, including SaCas9 and SpCas9 target sites in introns 51 and 53 and in exon 53 (Fig. 1b). Deleting exons 52 and 53 is one of the applied therapeutic strategies by Bengtsson et al.^6^. We wondered whether we could detect AAV sequences when the large ~45 kb genomic DNA region containing exons 52 and 53 is deleted; thus we performed a PCR with primers in introns 51 and 53. Sequencing this PCR product revealed the anticipated 45 kb deletion, but also high levels of AAV integration between the cut sites (Fig. 1b, and Supplementary Data 3) with up to 47.5% efficiency.

Altogether these results confirm that AAV integration is a common occurrence after Cas9-induced breaks in vivo in brain, cochlea and muscle.

### Genome editing events after AAV-mediated CRISPR delivery

Next, we set out to more thoroughly characterize the molecular outcomes of genome editing and AAV vector integration in the treated hippocampus samples. Analysis of the size of indels occurring at the *Dnmt3b* on-target site revealed a bimodal distribution of relatively small deletions and longer insertions (Fig. 1c–e). While the majority of indels were relatively small (< 25 nt) in size (92.3 ± 1.4%), we also observed insertions longer than 25 nt (7.3 ± 1.6% of all indels; Fig. 1d, e). By aligning the long insertion reads to the AAV genome, we identified that these larger insertion events were AAV vector integrations (Fig. 1e). The vast majority (97.8%) of the junction sites between *Dnmt3b* and AAV were within 1 bp of the CRISPR cut site (3 bp upstream of the protospacer adjacent motif (PAM) site), suggesting that this AAV integration was indeed at the expected CRISPR-induced break site (Fig. 1e). Additionally, the majority (83.5%) of AAV-genomic junctions contained elements of the viral ITRs (Fig. 1e, f). However, it was not possible to determine the length and efficiency of AAV vector genome integration events due to the relatively short NGS read length (i.e., longer integrants containing ITR and other components of the AAV cassette may not be detected or detected at lower frequencies (Fig. 1f). By examining the genomic location of small indels and AAV-genomic junction sites, we confirmed the two types of events overlap suggesting that AAV integrates precisely at the cut site, but not around it (Fig. 1g). Furthermore, these results show that the majority of AAV-genomic junctions contain sequences of the ITR elements in vivo.

### Genome-wide AAV mapping from mouse brain

Next, we wondered whether the expression of Cas9 and/or gRNA would facilitate AAV integration into genomic sites outside of the target locus. To map AAV integrations within the genome in the brain, we injected mice into the hippocampus with AAV1 vectors encoding for Cas9 and/or gRNA and performed deep sequencing of AAV-genomic junctions. To locate AAV integration sites, we used a modified version of the GUIDE-Seq^24^ pipeline adapted for AAV ITRs with primers specific for the ‘*a*’ region (Supplementary Fig. 1). We injected mice with AAV1 vectors encoding Cas9 and gRNAs targeting *Mecp2*, *Dnmt3b*, and *APP*^*SW*^ sequences and 6 weeks after injection, we collected the hippocampus and isolated genomic DNA.

Integration sites were mapped with Virus-Clip^25^ and output was filtered to exclude false positive hits. We excluded sites that showed homology to the vector sequence or sites that only showed genomic sequences, but no viral ITR elements (for full details see Methods section). For a full list of all the sites, including excluded sites and reasons for exclusion, see Supplementary Data files 4–9. For all true integration sites showing genomic alignments see Supplementary Data 10–15 and Supplementary Data 16.

**Fig. 2:**
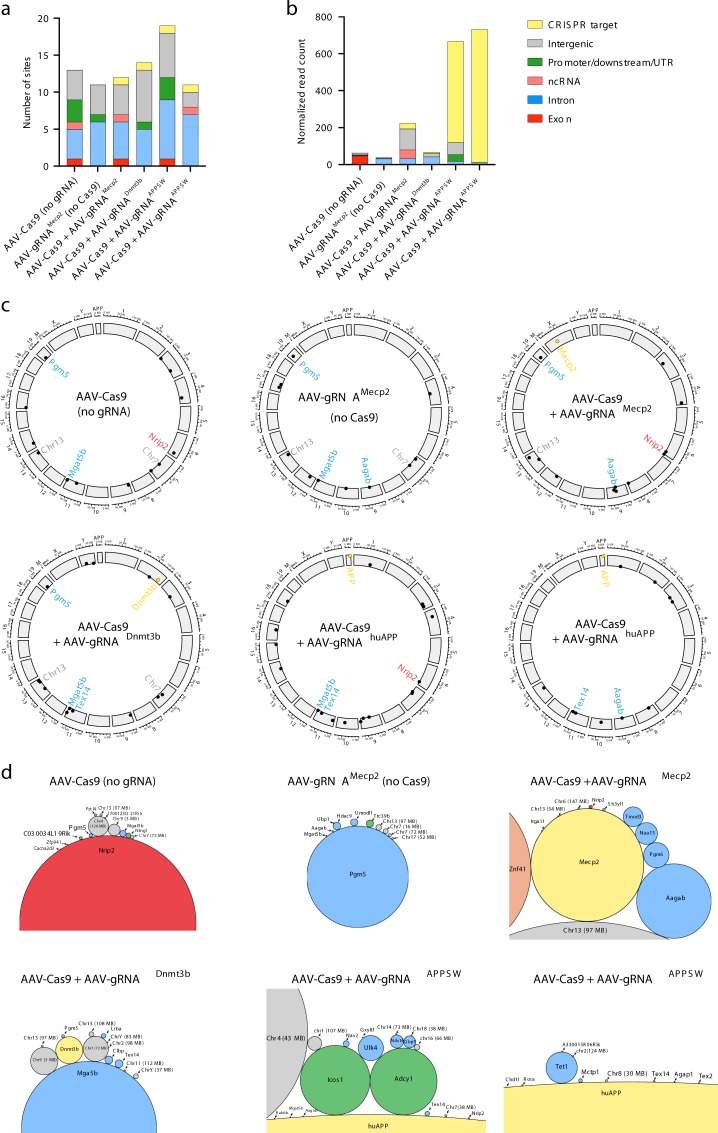
Genome-wide AAV mapping from CRISPR treated mouse brains. **a** Total number of unique integration sites. In the case of AAV-Cas9, AAV-gRNA^Mecp2^, AAV-Cas9 + gRNA^Mecp2^, and AAV-Cas9 + gRNA^Dnmt3b^, three mouse brains were pooled together for library construction. For AAV-Cas9 + gRNA^APPSW^, hippocampus tissues from two animals were separately processed for library construction. The colors represent different genomic integration types and are based on the output of Virus-Clip. **b** Total number of reads that contain integrants normalized to total reads, based on the output from Virus-Clip. **c** Circos plots on showing the chromosomal location of AAV integration events. The more eccentric a dot is, the higher the normalized read count for that site is, on a logarithmic scale. The gene names inside the circle represent either CRISPR targets or sites that are common integration events (present at least in three different samples). Colors of gene names are the same as in **b**. The human APP gene was added as a separate chromosome. **d** Bubble-plots showing all integration sites. The size of the circle is proportional to the normalized read count. The color was kept consistent in the figure in respect to type of integration. Intergenic integrations are marked by the chromosome and location. Source data are provided as a Source Data file

First, we analyzed the global integration profile of AAV in all samples and identified 11–19 unique integration sites per condition. We did not observe an increase in the overall number of integration sites when Cas9 and gRNA were both present (Fig. 2a). The majority of the integration sites were found to be intronic or intergenic, with an average of 44.7% and 33.4%, respectively. Exonic and regulatory region (promoter, downstream) integrations were rather rare (3.5% and 9.2%, respectively). In mice co-injected with AAV-Cas9 and AAV-gRNA vectors, we observed AAV integration in all three CRISPR target sites. Next, we analyzed the total integrant read counts normalized to the total number of sequenced reads (Fig. 2b). Total integration efficiency was the highest in animals treated with AAV-Cas9 and AAV-gRNA against *APP*^*SW*^ (Fig. 2b, 82.0% and 92.4% of integrants were found in the on-target region). This is not surprising as *APP*^*SW*^ is a transgenic mouse with multiple copies of the human transgene in a mouse genome. In the case of *Mecp2* and *Dnmt3b* targets, 13.4 and 10.7% of integrants were in the on-target locus. Importantly, AAV integration also occurred in AAV-Cas9 only and AAV-gRNA only conditions with a similar level to the AAV-Cas9 + AAV-gRNA^Dnmt3b^ condition.

Finally, we plotted all integration sites from all conditions and analyzed whether we could identify common sites. Integration sites appeared very variable and not consistent even between the two animals treated with AAV-Cas9 and AAV-gRNA^APPSW^ (Fig. 2c, d). However, we identified sites that favored AAV integration (Fig. 2c). These included *Mgat5b* (intronic, detected in 4/6 conditions), *Pgm5* (intronic, detected in 4/6 conditions), a region of chr13 (97 MB) (intergenic, detected in 4/6 conditions), *Aagab* (intronic, detected in 3/6 conditions), a region of chr7, 72 MB (intergenic, detected in 3/6 conditions), *Nrip2* (exonic, detected in 3/6 conditions), and *Tex14* (intronic, detected in 3/6 conditions) (Fig. 2c, d). None of the identified sites showed homology to the gRNA target region, suggesting that AAV integration into predicted off-target cut sites was below the level of detection.

Taken together, these results suggest that the presence of Cas9 alone or the presence of Cas9 and gRNA co-delivered by AAV does not influence the genome-wide integration efficiency of AAV (compared with the AAV-gRNA alone vector), except at the CRISPR target site.

### A miniaturized AAV allows characterization of CRISPR DSBs

**Fig. 3:**
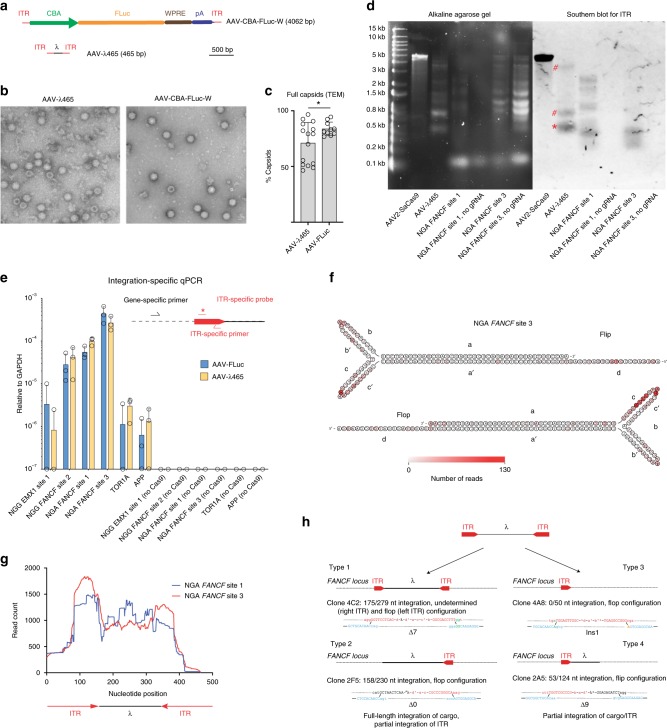
Characterization of AAV vector integration into CRISPR cut sites using a miniaturized AAV genome. **a** Schematic of standard-sized AAV-CBA-FLuc vector (top, 4062 bases) vs miniaturized AAV-λ465 (bottom, 465 bases). Chart is to scale. **b** Transmission electron microscopic examination of iodixanol gradient-purified capsids of AAV2-λ or AAV2-CBA-FLuc. **c** quantitation of full vs empty capsids (bars represent mean ± SEM, data from two independent experiments, 15 and 10 images were taken and 954 and 1231 capsids were counted for AAV-λ465 and AAV-CBA-FLuc, respectively, and *p* = 0.0254, unpaired *t*-test). **d** Alkaline gel electrophoreses and Southern blot for AAV genomes from iodixanol purified vectors (AAV2-CMV::NLS-SaCas9-NLS-3xHA-bGHpA;U6::BsaI-sgRNA (pX601, 4.8 kb size) and AAV-λ465 (465 bp size) and cellular genomic DNA containing integrated AAV-λ465. For Southern blot, we used a probe specific for the ITR region. Star (*) highlights the 465 bp expected band and pound (#) sign highlights concatemers in the AAV-λ465 genome. **e** ITR-genomic fusion events quantified by integration-specific qPCR assay, using AAV-λ465 or AAV2-CBA-FLuc vectors determined (bars represent mean ± SD). Three independent experiments were performed using two technical replicates each. **f** Heatmap of AAV specific ITR nucleotide integration at CRISPR cut site. More saturated red indicates higher frequency of breaks at the given position. **g** Integration profile of the entire miniaturized AAV genome from U2-OS cells. **h** Representative individual AAV integration clones showing different forms of integration detected (for all the clones, see Supplementary Fig. 4). Source data are provided as a Source Data file

The profile of AAV integration in CRISPR cut sites determined by NGS appears to favor the ITR region. However, due to the limitations of the read length of NGS (the size of the Cas9 encoding vector is over 4 kb and the gRNA vector over 2 kb), the profile generated in Fig. 1f is likely to be biased toward the periphery of the AAV vector genome, where ITRs are located. Thus, the question of whether AAV integration is occurring preferentially in the ITR region, or whether the AAV vector integrant is full length or fragmented, could not have been determined. In order to overcome this issue, we designed a minimal AAV construct, in which a very short cargo (175 bp) is flanked by ITR elements (Fig. 3a). For stuffer DNA, we chose a region of the λ-bacteriophage genome which is highly orthologous to the human/mouse genome. Together with the ITR elements, we synthesized and cloned this short vector, termed AAV-λ465 (465 bp).

First, we asked whether such a short AAV transgene cassette could be packaged into AAV2 capsids during production in 293T cells. Cells were transfected with plasmids required for AAV2-λ465 generation. As a control, we used a 4 kb long AAV vector encoding firefly luciferase (FLuc) driven by the chicken beta actin promoter (CBA), AAV-CBA-FLuc. First, we performed qPCR to quantitate the amount of each vector in the cell culture media using the ITR regions for probing. Titers of DNase-resistant AAV particles of AAV2-λ465 and AAV2-CBA-FLuc were not significantly different, suggesting successful packaging of the small genome into AAV2 capsids (AAV2-λ465: 7 × 10^12^ ± 3.3 × 10^12^ vg/mL (mean ± SD) and AAV2-CBA-FLuc: 7.4 × 10^12^ ± 6.6 × 10^12^ (mean ± SD), difference not significant by *t*-test). Next, we isolated and purified AAV-λ vectors on iodixanol gradients, and achieved DNase-resistant AAV particle titers of > 10^12^ vg/ml. We performed transmission electron microscopy (TEM) on AAV2-λ465 and AAV2-CBA-FLuc (Fig. 3b). AAV capsids were observed both in AAV2-λ465 and AAV2-CBA-FLuc samples. We also counted full vs. empty capsids on TEM images and observed that AAV2-λ465 has significantly higher empty capsids (28.8%) compared with AAV2-CBA-FLuc (16.1%); unpaired *t*-test, *p* = 0.0254, Fig. 3c). The DNase-resistant vector titers as well as TEM analysis of capsids suggests successful packaging of AAV2-λ465. Due to the small size of the *λ* cassette, we wondered if individual AAV2 capsids would package multiple miniaturized monomeric genomes. If there would be on average multiple genomes per capsid, one would expect lower number of capsids for a given amount of AAV genomes. To determine capsid/genomic copy ratio, we performed ELISA for AAV2 capsids and qPCR for AAV genomes. Capsid/genomic copy ratio was not significantly different for AAV2-λ465 and AAV2-CBA-FLuc at 10^9^ vg/well, and was significantly increased (unpaired *t*-test, *p* = 0.026) in the case of AAV2-λ465 compared with AAV2-CBA-FLuc at 10^10^ vg/well (Supplementary Fig. 2). These results suggest that AAV2-λ465 on average does not harbor more than one monomeric genomic copy per capsid as compared with a full-length AAV. However, concatemeric AAV-λ465 genomes could potentially be packaged into a given capsid, which may not be detected by the ITR-specific qPCR used to titer the vector preparation, due to ITR recombination during concatamerization. To ascertain this possibility, we ran purified AAV2-λ465 or purified AAV2-Cas9 (full-length control vector) on an alkaline agarose gel and stained the gel to observe the size of the genomes. As expected the full-length AAV2-Cas9 vector ran at ~5 kb. For AAV2-λ465, a band migrating just below 0.5 kb indicating monomeric genomes was observed. Interestingly, additional bands between ~0.7 and 4 kb were observed indicating some packaged genomes were concatemers (Fig. 3d). A Southern blot using an ITR-specific probe confirmed the presence of monomeric and concatemeric genomes in the packaged AAV capsid (Fig. 3d). Importantly, we did not observe smaller bands in the case of AAV2-λ465, suggesting no significant fragmentation of the small AAV genome.

To precisely quantify and characterize AAV integration into nuclease-induced breaks in the U2-OS human cell line using AAV2-λ465 (or AAV2-FLuc for comparison), we supplied Cas9 and gRNA using transfected plasmids into cells after overnight transduction with the AAV vector. First, we analyzed indel formation and AAV integration in four target sites (Supplementary Table 1). All target sites that showed indel formation showed AAV capture as well, as assayed by NGS (Supplementary Table 1). We observed AAV capture rates between 3 and 38% in the case of AAV2-λ465 (Supplementary Table 1). Next, we developed an integration-specific qPCR assay with one primer being in the ITR and one primer being in the target gene (Fig. 3e). We selected the ITR region as the priming site as the ITR was present in the junction in the vast majority on fusion reads (Fig. 1f). We assessed the presence of ITR-genomic fusion events in the case of six different genes (Fig. 3e). There was no amplification from cells treated with Cas9 plasmid and AAV vector only (i.e., no gRNA) (Fig. 3e) indicating that a site-specific DSB is required for AAV integration. In contrast, the integration-specific qPCR assay showed integration in all target genes when cells were transfected with Cas9 and gRNA plasmids, and co-transduced by AAVs. There was no difference between the *GAPDH*-normalized ITR-genomic fusion events between the 465 bp AAV2-λ465 and the 4.1 kb AAV2-FLuc construct in any of the target genes analyzed (Fig. 3e).

Since we observed concatemeric genomes packaged in AAV2-λ465, we analyzed integration of concatemers in U2-OS cells using alkaline gel electrophoresis and Southern blotting. Using a probe specific for the ITR region, analysis of genomic DNA from U2-OS cells treated with Cas9/gRNA and AAV2-λ465 revealed integration into NGA *FANCF* site 1 and NGA *FANCF* site 3 (NGA is the PAM site of the SpCas9-VRQR PAM variant^1^), but there was no integration in the no gRNA control (Fig. 3d). The major integrant size was smaller than the AAV2-λ465 genome, but we observed larger bands (up to 3 kb) particularly in the case of NGA *FANCF* site 1, indicating concatemeric integration of AAV-λ465.

Next, to analyze if there is a preferred breakpoint in the ITR region we determined, for each nucleotide in the ITR region, how frequently that nucleotide was represented in fusion reads at the breakpoint (i.e., breakpoint would be adjacent to this nucleotide). A heatmap (Fig. 3f) revealed that there are preferential breakpoints in the “b” and “c” loop regions of ITR elements. Next, we analyzed AAV2-λ465 integration into the *FANCF* NGA sites 1 and 3^24^ using a specific gRNA, to assess whether there were any regions of the ITR which are present more often at the indels. Interestingly, in both the “flip” and “flop” ITR conformations, all ITR regions were present at relatively similar read counts (Supplementary Fig. 3). We observed a high frequency representation of the ITR elements when plotting the coverage of AAV2-λ465 (Fig. 3g), although we also readily detected integration of the λ sequence. Finally, in order to precisely characterize integrants and indels at the same time, we cloned amplified PCR fragments into a vector and performed Sanger sequencing of 285 individual bacterial clones from both sides of the integrant. We were able to recover 20 clones with AAV vector sequences. Integration of the λ sequence was either full length or partial (Fig. 3h). We observed four major integration types at the NGA *FANCF* site 3 locus: (Type 1;2 clones recovered) both ITRs are present and the full λ payload is detectable; (Type 2;4 clones recovered) full AAV-λ cargo with one ITR and no second ITR; (Type 3; 5 clones recovered) ITR-only integrations; and (Type 4;9 clones recovered) one ITR with partial λ sequences (Fig. 3h and Supplementary Fig. 4). We did not detect any integrants lacking ITR sequences. The presence of microhomology regions (depicted as green nucleotides on Fig. 3h and Supplementary Fig. 4) were sometimes present, however, for several clones, no microhomology regions were observed. These data suggest that the ITR is required for integration, but only one ITR is needed for a successful capture. Furthermore, our data suggest that the majority of integration events do not contain the full-length AAV genome, however, full-length AAV integration events are also readily detected.

## Discussion

Genome editing as a therapeutic tool is developing at a rapid pace, with the first in vivo clinical trials being planned^11^ or underway (clinicaltrials.gov: NCT03041324, NCT02702115). The discovery of genome editing systems allows specific targeting of genomic sequences, although gene editing outcomes are generally less predictable and are probably cell-type specific. A DSB induced by CRISPR elements will be followed by cellular DNA repair, including NHEJ, microhomology-mediated end joining (MMEJ) or homology directed repair (HDR)^26,27^. The presence of various DNA repair machineries and the error-prone nature of NHEJ leads to genomic heterogeneity after gene editing. It is crucial to better understand and predict gene editing outcomes in preclinical studies in order to design as safe clinical trials as possible.

In this study we set out to analyze one potential outcome of gene editing: AAV vector integration. AAV is a virus that is generally maintained in an extrachromosomal form. Wild-type AAV is known to integrate into the *AAVS1* site of the human genome, however, this is mediated by the Rep protein and AAV vectors used in gene therapy lack the Rep protein^28,29^. However, even AAV vectors can integrate at a low frequency (~0.1% of total vector genomes), and studies conducted in one strain of mice found AAV insertion into the *Rian* locus associated with hepatocellular carcinoma formation^30^. This locus, however, is absent in non-human primates (NHPs) and humans, and hepatocellular carcinoma induction has not been associated with AAV vectors in NHPs and humans^31^. There is accumulating evidence of AAV vector genome integration into nuclease-induced breaks after radiation^32^, zinc-finger nucleases^14^ or CRISPR/Cas9 nucleases^9,15^ In this study we also show AAV integration into Cas9-induced breaks in vitro and in vivo in therapeutically relevant genes. When using a long extension time to allow for PCR amplification of large integrants, AAV capture efficiencies were found to be up to 47%. We observed similar integration profiles and AAV capture rates in brain, cochlea and muscle. This suggests that AAV integration is a major gene editing outcome and has to be considered in gene editing applications. Previous studies have suggested the role of ITRs in this process of integration^12^ and our study confirmed this finding. Using NGS, we were able to identify the nucleotides in the ITR loop regions that are most likely to serve as breakpoints.

We next focused on AAV-Cas9/AAV-gRNA targeting therapeutically relevant genes in the brain and asked whether CRISPR can enhance genome-wide AAV integration. AAV sequencing revealed integration of the AAV cassette into all target genes analyzed (*Dnmt3b*, *Mecp2*, and *APP*^*SW*^), as expected. We did not observe a significant increase in overall AAV integration when using Cas9 compared with no Cas9 present (AAV-gRNA only). This suggests that AAV-mediated expression of Cas9 does not mediate a significant genome-wide genotoxicity. This is in line with recent data showing that appropriately designed gRNAs do not mediate off-targets in vivo^33^. Interestingly we found short sequences in some target genes that have homology to the ITR region. The integration sites detected in the brain were different from what it has been reported in the mouse liver^34^ suggesting that integration is cell- or tissue specific.

Previous studies could not demonstrate that AAV integration involves the full length or only partial integration of the vector, as amplifying and sequencing through a ~3–5 kb AAV cargo is difficult. To overcome this hurdle, we synthesized a very short AAV (465 bp), AAV-λ465, which could be sequenced entirely after integration. This miniature AAV cassette packaged efficiently in AAV2 capsids, as assessed by qPCR titering of DNase-resistant vector particles, with an only slightly elevated fraction of empty capsids. Southern blotting of purified vector, however, revealed both monomeric 465 bp vector as well as concatemers. We observed a band at ~700 bp probably corresponding to doublets with processed (shorter) ITR elements, but also larger concatemers up to 4 kb.

Next, we analyzed integration of AAV-λ465 with qPCR, NGS, Southern blotting and TA cloning. An integration-specific qPCR analysis confirmed that a CRISPR-mediated double-stranded break is required for integration. We observed no difference in ITR-genomic fusion events between AAV-λ465 and a full-length AAV using qPCR suggesting similar integration efficiency. One could speculate that these data indicate that AAV size is not a major determinant of integration efficiency but is dependent on the presence of ITR. However, a comparison between AAV-λ465 and a full-length AAV regarding integration efficiency is difficult due to the presence of heterogenous genomes in the minimal AAV vector. Southern blotting of genomic DNA of U2-OS cells revealed integration events of various sizes after AAV-λ465 transduction, including shorter and longer integrants than the size of the AAV-λ465 plus the genomic region. The shorter integrants likely correspond to fragmented AAV genomes also observed by TA cloning. The larger integrants likely represent concatemers formed either by integration of the concatemeric genomes or concatemerization of vector genomes after transduction. Despite the heterogeneous nature of AAV-λ465, however, it did effectively characterize the integration pattern of monomeric AAV genomes, which prior studies had not achieved due to the challenges of sequencing full-sized AAV genomes.

Although standard-sized AAV vectors would not form large concatemers within a capsid, concatemerization of AAV genomes does occur during transduction. This has been well characterized in prior studies as episomal forms of AAV^35–37^. To our knowledge, no groups have analyzed integration of concatemers of AAV into the genome using NGS. While our genome-wide analysis did not assay for this form of integrant, it will be interesting to assess in future studies of AAV vector integration into the genome.

TA cloning of integrated AAV fragments showed various types of integration of the AAV-λ465 cassette. We observed full-length integration of the cargo, fragmented integrations and also ITR-only integrants. These fragments are likely not coming from input fragmented AAV genomes, as Southern blotting of purified vector did not reveal any fragmented genomes for AAV-λ465. Concatemers were not detected with TA cloning, however, this is probably due to the fact that PCR and TA cloning are biased toward smaller fragments. Most importantly, TA cloning did not detect any integration of the cargo sequence without the presence of at least one ITR. TA cloning also revealed indel formation in the genomic DNA indicating the activity of NHEJ machinery. The requirement of short homology regions between ITRs and genomic target regions was not apparent, with several clones lacking these short homology regions. This suggests that integration can happen independent of the repair machinery. The TA cloning results suggest a hypothesis of integration: (1) after double-stranded break, one broken ITR is captured at specific bases, (2) the other end of the AAV vector is chewed back, and (3) the remaining AAV vector fragment is ligated to the other end of the genomic DNA. In some cases, the two ITRs get captured on either end of the break leading to full-length AAV integration. The loss of nucleotides from the genomic DNA is commonly seen suggesting NHEJ mediated repair. The integration of AAV vectors at DSBs induced by CRISPR is likely to occur via similar mechanisms to that described by the seminal work of David Russell’s group which showed AAV ITR mediated integration into DNA-damage induced DSBs^32^. Our work, similar to this prior study, observed ITR sequences present at DSBs with some clones showing microhomology between ITR and genomic DNA and others with no apparent homology. Similarly, both studies demonstrated apparent hot spots within the loop regions of the ITRs which were found at breakpoints.

During the course of our work, a very recent publication by Nelson et al.^15^ described long-term efficacy of CRISPR editing for muscular dystrophy in a mouse model, and the authors also reported AAV sequences integrated at the CRISPR cut site. Our data confirm AAV integration into CRISPR-induced double-stranded breaks in other organs, including brain and cochlea. There are two major features that differentiate our work and that of Nelson et al.^15^. First, our use of the miniaturized AAV vector allowed us to characterize the complete profile of the AAV integration events, which had not been possible before with standard AAV where the genome size and the limits of next-generation sequencing prevented this from being achieved. Second, our study provides a genome-wide analysis of AAV integration in the context of CRISPR in the brain.

Taken together, we have shown that AAV integration at the on-target cut site is a major gene editing outcome in vivo and it has to be considered when assessing safety. Importantly, however, we did not detect increased genome-wide integration of AAV in the presence of CRISPR/Cas9 in the brain suggesting high target specificity. Furthermore, AAV integration could be used as a sensitive assay to detect the presence of double-stranded breaks, and, in the future, it might be applied to directly assaying genome-wide specificities of genome editing nucleases.

## Methods

### Plasmids and cloning

pX551 was a gift from Feng Zhang (Addgene plasmid # 60957). pX552 was a gift from Feng Zhang (Addgene plasmid # 60958). pX601 was a gift from Feng Zhang (Addgene plasmid # 61591). Supplementary Table 2 has information on the plasmids used in this study. Cloning of gRNAs (Supplementary Table 3) into pX552 was performed with FastDigest LguI (Thermo Scientific) and verified by Sanger sequencing using a U6 sequencing primer: 5′-GACTATCATATGCTTACCGT-3′. To create AAV2-λ465, GenScript (Piscataway, NJ) synthesized the λ fragment using sequence from Enterobacteria phage lambda, (NC_001416.1) flanked by KpnI and SphI sites (for the sequence see Supplementary Data 17). We cloned two different length fragments into a single stranded AAV-CBA-W empty backbone cut with KpnI and SphI. The final vector has only a short linker, the sequence of two GUIDE-Seq oligos and a 70 nt fragment from the λ phage (Supplementary Data 17). The total length of the AAV vector including the two ITRs on either end were 465 bp. Cloning was validated by running KpnI/SphI digested vector on a gel and Sanger sequencing.

### AAV production

For production of AAV vectors, we used the triple transfection method. Briefly, we plated ten 15-cm tissue culture dishes with 1.5 × 10^7^ HEK-293T cells (ATCC® CRL-3216) per dish. The next day, cells were transfected using the calcium phosphate method, with the adenovirus helper plasmid (pAdΔF6, 26 µg), rep/cap plasmid (e.g., AAV2 or AAV1, 12 µg), and ITR-flanked transgene cassette plasmid (10 µg), per plate, to induce production of AAV (pX551, pX552, AAV-λ465, AAV-CBA-FLuc-WPRE). The day after transfection, medium was changed to DMEM containing 2% fetal bovine serum (FBS). AAV was purified from the cell lysate using iodixanol density-gradient ultracentrifugation. Buffer exchange to PBS was conducted using Zeba spin desalting columns (7 K MWCO; Thermo Fisher Scientific) and further concentration was performed using Amicon Ultra 100-kDa MWCO ultrafiltration centrifugal devices (Millipore). Vectors were stored at 80 °C until use. We quantified AAV vector genomes in AAV preparations using TaqMan qPCR with primer and probes specific to the ITR region of the virus.

### Transmission electron microscopy

Five microliters of the AAV sample was adsorbed for 1 min to a carbon-coated grid (EMS) that had been made hydrophilic by a 20 s exposure to a glow discharge (25 mA). AAVs were stained with 1% uranyl acetate (EMS catalog # 22400) for 20–30 s. After removing the excess stain with a filter paper, the grids were examined in a TecnaiG² Spirit BioTWIN and images were recorded with an AMT 2k CCD camera at the Harvard Medical School Electron Microscopy Facility.

### Capsid ELISA

ELISA plates were coated with A20 anti-AAV2 antibody (0.1 μg/well) in bicarbonate buffer overnight. Plates were washed twice with PBS, then blocked blocking solution (Thermo, Casein blocking solution in TBS: Blocker Casein in TBS, 100 mL, 1% (w/v) casein (Hammarsten grade) in 25 mM Tris, 150 mM NaCl, pH 7.4 containing Kathon Anti-microbial Agent, cat no: 37583) for 1 h at 37 °C. In total, 1 × 10^9^ and 1 × 10^10^ vector genomes of AAV-λ465 and AAV-CBA-FLuc were added to the plate in 100 μL volume and plates were incubated for 90 min at 37 °C. After washing three times with PBS-Tween, samples were incubated in the presence of a 1:20 dilution of primary antibody (Anti-AAV2, intact particles, clone A20, BIOTIN, American Research Products, Waltham, MA; Product Code: 03-61055B) in 50 μL volume. After washing three times with PBS-Tween, 1:2000 streptavidin-HRP was added (GE Healthcare, RPN1231V) and plates were incubated for 1 h at 37 °C. After the addition of TMB substrate, and H_2_SO_4_, absorbance was read at 450 nm. Samples were run in duplicates.

### Cell culture and nucleofection

U2-OS cells (Homo sapiens bone osteosarcoma, ATCC® HTB-96™) were cultured in Dulbecco’s modified Eagles media (DMEM) supplemented with 10% bovine serum albumin und 1% penicillin/streptomycin. We performed Mycoplasma testing on a monthly basis using the MycoAlert Kit (Lonza). Cells were transfected with the Nucleofector device (Lonza) using CM-104 program and the SE kit. For each transfection, we used 500 ng Cas9 plasmid and 250 ng gRNA plasmid (see Supplementary Table 2 for plasmid specification for each gene).

### TA cloning

U2-OS cells were treated with AAV-λ465 and were transfected with SpCas9-VRQR variant and gRNA against NGA *FANCF* site 3 (see details of transfection above). Genomic DNA was isolated by the Qiagen Blood and Tissue Kit, and 100 ng DNA was subjected to targeted PCR (see primers in Supplementary Table 4) using the Phusion polymerase (NEB). The samples were subjected to gel purification (gel was cut above the expected 132 bp PCR band and below ~600 bp). After gel purification, DNA sample was ethanol precipitated for 20 min at −20 °C and 1 μL reaction (12 ng) was used to in the TA cloning reaction according to manufacturer’s instructions (TOPO® TA Cloning Kit for Sequencing, ThermoFisher). Three full plates (285 clones) were sequenced with the T7 primer included in the cloning kit.

### Neuronal culture

In this study, we performed original experiments and for some cases we reanalyzed existing sequencing data from our previous publications. A detailed description of every data point is found in the Source Data file. For this study, wild-type C57BL/6 or C57BL/6:SJL male mice (see Source Data file for details) were housed with wild-type females for 3 days to generate timed pregnancies for embryo-derived, primary neuronal cultures^8^. At embryonic days 14–17, the females were euthanized by CO_2_ and the embryos were collected. After dissection, the embryonic cortices were collected separately in Hank’s Balanced Salt Solution with 1% penicillin (Pe)/streptomycin (St) and 10 mM HEPES buffer, pH 7.4 (HBSS) and placed on ice. The cortices were centrifuged at 200 × *g* for 5 min and dissociated in 500 µL Plating media (Neurobasal media—with 1% Pe/St, 10 mM HEPES buffer, 1xGlutaMAX, and 10% fetal bovine serum (FBS). Approximately 300,000 cells were plated in each well of 12 well, tissue treated, plastic plates (Corning, Inc.) coated with Poly-L-ornithine (1:4 in H_2_O, Sigma) and laminin (1:1000 in PBS). After 5 h, the media was replaced with Neurobasal media, similar to the plating media but containing serum-free B-27 supplement instead of FBS. At day 3, the neurons were treated with AAV1-pMecp2-SpCas9-spA (referred to as AAV-Cas9) mixed with AAV1-U6sgRNA(SapI)_hSyn-GFP-KASH-bGH (referred to as AAV-gRNA). We added 10^5^ genomic copies per cell with a 1:2 AAV-Cas9 to AAV-gRNA ratio. Control cells were transduced with AAV-Cas9 alone without gRNA. DNA was isolated as described at 21 days in vitro (DIV). All products for neuronal culture were purchased from Thermo Fisher Scientific if not otherwise stated.

### Animals

In this study, we performed original experiments and for some cases we reanalyzed existing sequencing data from our previous publications. A detailed description of every data point is found in the Source Data file. For this study, 8–10-week-old male C57BL/6 mice were used in this study. Animals were purchased from Charles River Laboratories. We attest that we have complied with all relevant ethical regulations for animal testing and research. The research described in this study was approved by Animal Care Committee and Partners Institutional Biosafety Committee of Partners Healthcare (Massachusetts General Hospital, Boston, MA, USA).

### In vivo injections and DNA isolation

Mice were anesthetized by intraperitoneal (i.p.) injection of 100 mg/kg ketamine and 10 mg/kg xylazine. A small burr hole was drilled in the skull and mice were stereotactically injected with a mixture of AAV1-Mecp2-Cas9 (5 × 10^9^ vg) and AAV1-U6-gRNA^Mecp2^-hSyn-GFP or AAV1-U6-gRNA^Dnmt3b^-hSyn-GFP (3 × 10^9^ vg into the left ventral dentate gyrus (coordinates: anterior/posterior (*y*): −3.5, mediolateral (*x*): + 2.7, dorsal/ventral (*z*): −3). We injected using a Hamilton syringe fitted with a 30 gauge needle at a rate of 0.2 µl/min using a pump and controller (World Precision Instruments, Sarasota, FL). Six weeks after injection, genomic DNA was isolated directly from hippocampus using the Qiagen Blood and Tissue Kit (Qiagen). Briefly, we added 360 μL of ATL buffer and 40 μL of proteinase K to dissected hippocampus tissue. Samples were then digested for 5 min at 56 °C and were homogenized using disposable pestles. Next, samples were digested for another 10 min at 56 °C. After vortexing, we added 400 μL of the AL buffer from the kit and incubated the samples for another 5 min at 56 °C. After the addition of 400 μL of 100% ethanol, we purified the genomic DNA according to the manufacturers’ instructions.

### Targeted DNA Sequencing

Genomic DNA from U2-OS cells, cultured cortical neurons, or brain tissue was PCR amplified using Phusion polymerase (NEB) according to the manufacturer’s instructions. Importantly, we kept the extension time long (4:15 min) so that even several kilobases could be amplified. PCR products were visualized on a 1% agarose gel using GelRed (Thermo Fisher) and purified on a column (PCR Purification Kit, Qiagen). PCR primers are given in Supplementary Table 4. For muscle samples, we separately amplified the targeted region in intron 51, in intron 53 and exon 53, as described previously^6^. We also assumed the deletion of exons 52–53 and therefore we performed a PCR reaction with a primer in intron 51 (DS-i51, forward) and intron 53 (DS-i53, reverse). For targeted deep sequencing we used 500–1400 ng genomic DNA. Sequencing was performed at the MGH DNA Core (Sanger sequencing and CRISPR-sequencing service). Paired-end reads (150 bp) were generated on an Illumina MiSeq platform with a 100 K read depth per sample.

### Genome-wide AAV sequencing (AAV-Seq)

Samples were pooled from three animals for NGS library construction in the case of *Mecp2* and *Dnmt3b* groups. For the *APP*^*SW*^ group, we analyzed two animals separately. As controls, we included three animals injected with AAV-Mecp2-Cas9 vector only and AAV-gRNA^Mecp2^-hSyn-GFP vector only. To amplify AAV from the genome, we used the GUIDE-Seq method adapted to AAV ITR sequences. Primer sequences and illustrated ITR priming sites are depicted in (Supplementary Fig. 1).

### Targeted deep sequencing analysis

Targeted deep sequencing data was analyzed using CRISPResso (Accession code, GEO: GSE78729)^38^ as previously described. Briefly, reads were split into reads 1 and 2 and then merged using flash v1.2.11 with default parameters. Next, CRISPResso was run with the following parameters: CRISPResso -r1<fastq_file> --split_paired_end -w 5 -c <protein_coding_sequence> --ignore_substitutions -a <amplicon_sequence> -g<gRNA_sequence>. Substitutions were ignored. To analyze the number of AAV integrants, sequencing files were aligned to AAV vector sequences using bwa (version 0.7.17-r1188), sorted with Samtools (version 1.7) and the number of reads that had vector sequences were quantified.

### Analysis of genome-wide integration of AAV vector sequences (AAV-Seq)

In order to determine the extent of genome-wide AAV vector integration, DNA from mice injected with AAV-Cas9 and AAV-gRNA was sequenced paired-end on an Illumina MiSeq, with an average read depth of 3 million reads per sample. Samples were analyzed using a viral integration detection pipeline, Virus-Clip (version 1.0)^25^. Briefly, Virus-Clip maps FASTQ reads from sequenced samples to indexed viral genomes of interest, using bwa (version 0.7.17-r1188). It extracts CIGAR strings from this analysis to identify sequence reads with a mix of viral and non-viral DNA. These reads are then mapped to a BLAST database of the host genome (in this case, the current mouse genomic build GRCm38/mm10) to identify breakpoint regions containing both mouse and vector DNA, which are assigned as sites of viral integration. These sites are annotated using ANNOVAR (version 2018Apr16)^39^ and outputted. In this manner, sequence reads that are not fusion reads, that is, purely episomal AAV DNA, are excluded from analysis. Manual curation was then applied to exclude putative integration sites that feature any sequences common to both mouse and vector genomes. Default Virus-Clip run parameters were used. From the Virus-Clip output file, we manually analyzed all sites and excluded false hits. We observed that in all animals injected with AAV-Mecp2-Cas9, the promoter region of *Mecp2* was flagged as a false integration site. Similarly, the gene *Syne2* was present in all samples except the AAV-Mecp2-Cas9 group. This gene showed similarity to the hSyn promoter driving GFP expression in the gRNA vector. In the next step, putative integration sites were analyzed to exclude sites that show either no ITR integration or did not appear to be bona fide fusion reads. To avoid the inclusion of genome-only regions amplified by the primers, only those sites were considered for which we were able to detect unique AAV ITR regions outside of the primer binding sites fused with genomic DNA. Fusion reads between genomic DNA and AAV were visualized using Geneious Prime 2019.0.4 (Supplementary Data 16).

### Alkaline gel electrophoresis and Southern blotting

Two hundred microliters of volume containing AAV genomes (3 × 10^12^ vg for AAV2-pX601 and 2 × 10^11^ vg for AAV2-λ465) were purified using High Pure Viral Nucleic Acid kit (Roche, Boulogne- Billancourt, France). DNase I treatment was not required because the crude cell lysates are treated with Benzonase during AAV vector purification and it is sufficient to remove plasmid DNA^40^. PCR products of NGA *FANCF* site 1 and NGA *FANCF* site 3 were purified on a column (Qiagen). Alkaline gel electrophoresis was performed as described previously^41^ with some modifications. The gel was run for 225 min at 30 V and staining was performed with SYBR Gold Nucleic Acid Gel Stain at 1:10,000 dilution. Southern blotting was performed as described originally by Ed Southern^42^. Briefly, we soaked the gel in alkaline transferbuffer (0.4 M NaOH and 1 M NaCl) twice for 20 min. The blotting was performed overnight onto a positively charged membrane (Hybond-N+ (20 cm × 3 m) GE Life Sciences, RPN203B). Next, the membrane was neutralized using neutralization buffer (0.5 M Tris-Cl, 1 M NaCl, pH 7.2) for 15 min and blocked for 2 h using Denhardt’s solution (Thermo Fisher). To detect the ITR in AAV vectors and PCR products, we used a biotinylated ITR probe (5′-CACTCCCTCTCTGCGCGCTCG-3′, 2.3 μL of probe was used from a 100 mM stock solution). Hybridization was performed in 6× saline-sodium citrate buffer (SSC, 0.9 M NaCl, 0.9 M sodium citrate tribasic dehydrate). After washing twice in 2× and twice in 0.1× SSC buffer, the membrane was developed with a Chemiluminescent Nucleic Acid Detection Module Kit (Thermo) and imaged with an iBright Imaging System (Thermo).

### Statistics

We used GraphPad Prism 7.0 for Mac OS and OriginPro (2015) for statistical analysis. To compare means, we used an unpaired two tailed *t*-test (after Shapiro-Wilk normality testing) or paired *t*-test; *p* values < 0.05 were accepted as significant.

### Reporting summary

Further information on research design is available in the Nature Research Reporting Summary linked to this article.

## Supplementary information

Description of Additional Supplementary Files

Supplementary Information

Supplementary Dataset 1

Supplementary Dataset 2

Supplementary Dataset 3

Supplementary Dataset 4

Supplementary Dataset 5

Supplementary Dataset 6

Supplementary Dataset 7

Supplementary Dataset 8

Supplementary Dataset 9

Supplementary Dataset 10

Supplementary Dataset 11

Supplementary Dataset 12

Supplementary Dataset 13

Supplementary Dataset 14

Supplementary Dataset 15

Supplementary Dataset 16

Supplementary Dataset 17

Supplementary Dataset 18

Reporting Summary

Source Data

## Data Availability

Raw sequencing files have been be uploaded to NCBI’s Sequence Read Archive (SRA, Bioproject ID: PRJNA563918). A list of uploaded files including SRA IDs are listed in Source Data File. Detailed data analysis is available in the Supplementary tables and Supplementary Data published with this paper. The plasmid containing the AAV2-λ465 is available upon completion of a standard Material Transfer Agreement with The Massachusetts General Hospital. A reporting summary for this Article is available as a Supplementary Information file. The source data underlying Figs. 1a, 1b, 1d, 1f, 1g, 2, 3c, 3e, 3g, Supplementary Figs. 2 and 3 and Supplementary Table 1 are provided as a Source Data file. Any other raw data that support the findings of this study are available from the corresponding author.

## Code availability

All sequence analysis was run using publicly available programs, which are referred to and referenced in the paper along with run parameters where relevant. Quantification of integrant numbers was performed with a script attached to this paper as Supplementary Data 18, which also utilized publicly available software.
