# Supplementary material for: High levels of AAV vector integration into CRISPR-induced DNA breaks
Source: Nat Commun. 2019 Sep 30;10:4439. doi: 10.1038/s41467-019-12449-2 (PMC6769011; doi:10.1038/s41467-019-12449-2)
Supplement: Supplementary file 1 — Description of Additional Supplementary Files [file 41467_2019_12449_MOESM1_ESM.pdf]

Legends for Supplementary Data

Title: Supplementary Data 1.

Description: CRISPResso analysis of genomic DNA from cultured cortical neurons treated with AAV1-Cas9 + AAV1-gRNA, data are summarized on Fig. 1a.

Title: Supplementary Data 2.

Description: CRISPResso analysis of genomic DNA from mouse hippocampus. Mice were injected with AAV1-Cas9 + AAV1-gRNA, data are summarized on Fig. 1b.

Title: Supplementary Data 3.

Description: CRISPResso analysis of *dmd* gene after CRISPR targeting of introns 51 and 53. PCR was performed with a forward primer in intron 51 and a reverse primer in intron 53. This PCR will only yield a ~160-200 bp product, if the intervening ~45 kb DNA is deleted after two CRISPR cuts in introns 51 and 53. The exon skipped product contains several AAV sequences integrated (insertions are in red boxes). The dashed line shows the anticipated breakpoint, the sequence of the left side is intron 51, the right side is intron 53.

Title: Supplementary Data 4.

Description: Genomic sites of AAV integration, Cas9 only injection. Output file of Virus-Clip (unfiltered). Sites that show homology with vector sequences (e.g. Mecp2 upstream and Syne2 [homology to Synapsin promoter]) and sites for which no genomic-AAV fusion reads have been identified are marked in red (excluded from analysis). All other sites, in which fusion reads have been detected are shown in green. ITR homology sequences are listed.

Title: Supplementary Data 5.

Description: Genomic sites of AAV integration, gRNA only injection (AAV-gRNA^{Mecp2}-syn-GFP). Output file of Virus-Clip (unfiltered). Sites that show homology with vector sequences (e.g. Mecp2 upstream and Syne2 [homology to Synapsin promoter]) and sites for which no genomic-AAV fusion reads have been identified are marked in red (excluded from analysis). All other sites, in which fusion reads have been detected are shown in green. ITR homology sequences are listed.

Title: Supplementary Data 6.

Description: Genomic sites of AAV integration, Cas9 + gRNA^{Mecp2} injection (AAV-Cas9 + AAV-gRNA^{Mecp2}-syn-GFP). Output file of Virus-Clip (unfiltered). Output file of Virus-Clip (unfiltered). Sites that show homology with vector sequences (e.g. Mecp2 upstream and Syne2 [homology to Synapsin promoter]) and sites for which no genomic-AAV fusion reads have been identified are marked in red (excluded from analysis). All other sites, in which fusion reads have been detected are shown in green. CRISPR target is marked in yellow. ITR homology sequences are listed.

Title: Supplementary Data 7.

Description: Genomic sites of AAV integration, Cas9 + gRNA^{Dnmt3b} injection (AAV-Cas9 + AAV-gRNA^{Dnmt3b}-syn-GFP). Output file of Virus-Clip (unfiltered). Output file of Virus-Clip (unfiltered). Sites that show homology with vector sequences (e.g. Mecp2 upstream and Syne2 [homology to Synapsin promoter]) and sites for which no genomic-AAV fusion reads have been identified are marked in red (excluded from analysis). All other sites, in which fusion reads have been detected are shown in green. CRISPR target is marked in yellow. ITR homology sequences are listed.

Title: Supplementary Data 8.

Description: Genomic sites of AAV integration, Cas9 + gRNA^{APP} injection (AAV-Cas9 + AAV-gRNA^{APPSW}-syn-GFP, animal 1). Output file of Virus-Clip (unfiltered). Output file of Virus-Clip (unfiltered). Sites that show homology with vector sequences (e.g. Mecp2 upstream and Syne2 [homology to Synapsin promoter]) and sites for which no genomic-AAV fusion reads have been identified are marked in red (excluded from analysis). All other sites, in which fusion reads have been detected are shown in green. CRISPR target is marked in yellow. ITR homology sequences are listed.

Title: Supplementary Data 9.

Description: Genomic sites of AAV integration, Cas9 + gRNA^{APP} injection (AAV-Cas9 + AAV-gRNA^{APPSW}-syn-GFP, animal 2). Output file of Virus-Clip (unfiltered). Output file of

Virus-Clip (unfiltered). Sites that show homology with vector sequences (e.g. Mecp2 upstream and Syne2 [homology to Synapsin promoter]) and sites for which no genomic-AAV fusion reads have been identified are marked in red (excluded from analysis). All other sites, in which fusion reads have been detected are shown in green. CRISPR target is marked in yellow. ITR homology sequences are listed.

Title: Supplementary Data 10.

Description: Genomic sites of AAV integration, Cas9 only injection. Output file of Virus-Clip (filtered). Only sites that show unique AAV-genomic fusion reads are shown (green). ITR homology sequences are listed.

Title: Supplementary Data 11.

Description: Genomic sites of AAV integration, gRNA only injection (AAV-gRNA^{Mecp2}-syn-GFP). Output file of Virus-Clip (filtered). Only sites that show unique AAV-genomic fusion reads are shown (green). ITR homology sequences are listed.

Title: Supplementary Data 12.

Description: Genomic sites of AAV integration, Cas9 + gRNA^{Mecp2} injection (AAV-gRNA^{Mecp2}-syn-GFP). Only sites that show unique AAV-genomic fusion reads are shown (green). CRISPR target is marked in yellow. ITR homology sequences are listed.

Title: Supplementary Data 13.

Description: Genomic sites of AAV integration, Cas9 + gRNA^{Dnmt3b} injection (AAV-gRNA^{Dnmt3b}-syn-GFP). Only sites that show unique AAV-genomic fusion reads are shown (green). CRISPR target is marked in yellow. ITR homology sequences are listed.

Title: Supplementary Data 14.

Description: Genomic sites of AAV integration, Cas9 + gRNA^{APPSW} injection (AAV-gRNA^{APPSW}-syn-GFP, animal 1). Only sites that show unique AAV-genomic fusion reads are shown (green). CRISPR target is marked in yellow. ITR homology sequences are listed.

Title: Supplementary Data 15.

Description: Genomic sites of AAV integration, Cas9 + gRNAAPPSW injection (AAV-gRNAAPPSW-syn-GFP, animal 2). Only sites that show unique AAV-genomic fusion reads are shown (green). CRISPR target is marked in yellow. ITR homology sequences are listed.

Title: Supplementary Data 16.

Description: Alignment of AAV-genomic fusion reads to genomic sequences, visualized by Geneious. Grey nucleotides do not align to genome (align to ITR), while colored nucleotides align to the respective genomic sequence. Only fusion reads are visualized. Those reads in which mate 1 is viral and mate 2 is genomic are not visualized.

Title: Supplementary Data 17.

Description: AAV-λ465 sequence.

Title: Supplementary Data 18.

Description: Script to analyze the number of AAV integrants in PCR amplified samples.
