# Supplementary material for: High levels of AAV vector integration into CRISPR-induced DNA breaks
Source: Nat Commun. 2019 Sep 30;10:4439. doi: 10.1038/s41467-019-12449-2 (PMC6769011; doi:10.1038/s41467-019-12449-2)
Supplement: Supplementary file 2 — Supplementary Information [file 41467_2019_12449_MOESM2_ESM.pdf]

Supplementary Table 1.

Gene	gRNA	Indel%*	Integr%*	AAV Capture%**
NGG *EMX1* site 1	none***	<0.1%	0%	N.D.
NGG *EMX1* site 1	FYF1548	3%	0.10%	3%
NGG *FANCF* site 2	MSP815	3.30%	0.15%	4%
NGA *FANCF* site 1	MSP818	23.20%	14.10%	38%
NGA *FANCF* site 3	MSP820	32.50%	6.13%	16%

*as determined by targeted deep sequencing
**AAV capture was defined as AAV integration normalized to indels (AAV integration was also considered as an indel)
***no gRNA was used and indel and integration rates were determined at the *EMX1* locus

Supplementary Table 1. Indel percentages and AAV capture rates in U2-OS cells determined by targeted NGS. AAV capture percent was defined as AAV insertion normalized to all indel events (AAV integration was also considered as an indel event). Source data are provided as a Source Data file.

Supplementary Table 2. Plasmids used in this study.

Plasmid	Specification	Origin (reference)
pX551	pAAV-pMecp2-SpCas9-spA (AAV-SpCas9), Addgene ID: 60957	Swiech L et al, Nat Biotechnol 2014
pX552	pAAV-U6sgRNA(SapI)_hSyn-GFP-KASH-bGH, Addgene ID: 60958	Swiech L et al, Nat Biotechnol 2014
pX601	pX601-AAV-CMV::NLS-SaCas9-NLS-3xHA-bGHpA;U6::BsaI-sgRNA	Ran et al, Nat Biotechnol 2015
SQT817	CAG-humanSpCas9-NLS-3xFLAG, Addgene ID: 53373	
FYF1548	gRNA plasmid based on MLM3636 (NGG *EMX1* targeting), pUC19 backbone	Kleinstiver BP et al, Nat Biotechnol 2016
MSP815	gRNA plasmid based on MLM3636 (NGG *FANCF* site 2 targeting), pUC19 backbone	Kleinstiver BP et al, Nat Biotechnol 2016
BPK2797	CMV-T7-humanSpCas9-VRQR(D1135V, G1218R, R1335Q, T1337R)-NLS-3xFLAG	Kleinstiver BP et al, Nat Biotechnol 2016
MSP818	NGA gRNA plasmid based on MLM3636 (NGA *FANCF* site 1 targeting), pUC19 backbone	Kleinstiver BP et al, Nature 2015
MSP820	NGA gRNA plasmid based on MLM3636 (NGA *FANCF* site 3 targeting), pUC19 backbone	Kleinstiver BP et al, Nature 2015
MLM3636	gRNA plasmid backbone (empty vector), pUC19 backbone	Kleinstiver BP et al, Nature 2015

References:

Swiech L, Heidenreich M, Banerjee A, Habib N, Li Y, Trombetta J, Sur M, Zhang F. In vivo interrogation of gene function in the mammalian brain using CRISPR-Cas9. Nat Biotechnol. 2015 Jan;33(1):102-6.

Kleinstiver BP, Pattanayak V, Prew MS, Tsai SQ, Nguyen NT, Zheng Z, Joung JK. High-fidelity CRISPR-Cas9 nucleases with no detectable genome-wide off-target effects. Nature. 2016 Jan 28;529(7587):490-5.

Kleinstiver BP, Prew MS, Tsai SQ, Topkar VV, Nguyen NT, Zheng Z, Gonzales AP, Li Z, Peterson RT, Yeh JR, Aryee MJ, Joung JK. Engineered CRISPR-Cas9 nucleases with altered PAM specificities. Nature. 2015 Jul 23;523(7561):481-5.

Ran FA, Cong L, Yan WX, Scott DA, Gootenberg JS, Kriz AJ, Zetsche B, Shalem O, Wu X, Makarova KS, Koonin EV, Sharp PA, Zhang F. In vivo genome editing using Staphylococcus aureus Cas9. Nature. 2015 Apr 9;520(7546):186-91.

Supplementary Table 3. gRNAs used in this study

gRNA ID	Cas9	Target gene	gRNA sequence + PAM (5'-3')
pX552-mTmc1	SpCas9	mouse *Tmc1*	GGGTGGGACAGAACATCCCCAGG
pX552-Mecp2	SpCas9	mouse *Mecp2*	CCATTCTGCAGAGCCAGCAGAGG
pX552-Dnmt3b	SpCas9	mouse *Dnmt3b*	GAGAGGGTGCCAGCGGGTATGAGG
pX552-SW1	SpCas9	APP^{SW1}	GGAGATCTCTGAAGTGAATCTGG
FYF1548	SpCas9	NGG *EMX1* site 1	GAGTCCGAGCAGAAGAAGAAGGG
MSP815	SpCas9	NGG *FANCF* site 2	GCTGCAGAAGGGATTCCATGAGG
MSP818	SpCas9-VRQR	NGA *FANCF* site 1	GAATCCCTTCTGCAGCACCTGGA
MSP820	SpCas9-VRQR	NGA *FANCF* site 3	GCGGCGGCTGCACAACCAGTGGA
MLM3636 Tor1A	SpCas9	TOR1A	GATGACATTTTTCCCCAAAGAGG
MLM3636-APP	SpCas9	APP^{WT}	GGAGATCTCTGAAGTGAAGATGG

Supplementary Table 4. Oligo sequences used in the study
*note that this primer pair amplifies the cDNA of human APP^{SW} (exon 16) inserted in the mouse genome.

Target	Assay	Forward (5'-3')	Reverse (5'-3')
Tmc1	Targeted PCR	CCATCAAGGCGAGAATGAAT	TAAAGGGACCGCTCTGAAAA
Mecp2	Targeted PCR	GCAAGGTGGGGTCATCATAC	GCAAGCATGAGCCACTACAA
Dnmt3b	Targeted PCR	GTGCCCTGTCTGCCTCTTAC	ACCTCTGGTCTCTGGTGTGC
APP^{SW1}*	Targeted PCR	GGTAGGCTTTGTCTTACAGTG	CAGGATGAACCAGAGTTAATAGGT
Dmd intron 51	Targeted PCR	CACACATTTGTCCTTATGATTAAGATTTGG	GCAACAACACTCTTAAACACTGAG
Dmd intron 53	Targeted PCR	ATGTCCTTTGCCACCATGCTAA	GCATCTATTACTTTTCCTAAGAAGAAATT
Dmd exon 53 (outside primers)	Targeted PCR	GCTGAGGTAATAGAGCCAAG	CTGTGATCTTCTTTTGGATTGC
Dmd exon 53 (nested primers)	Targeted PCR	TTTCCACTGTCTTCTCTTGAGTAA	CTACTGTGTGAGGACCTTCTTTC
AAV_+_1	AAV-Seq	GGATCTCGACGCTCTCCCTGGAGTTGGCCACTCCCTCTCTG	
AAV_-_1	AAV-Seq	GGATCTCGACGCTCTCCCTCGAGCGAGCGCGCAGAGAGGGA	
AAV_+_2	AAV-Seq	CCTCTCTATGGGCAGTCGGTGATTCTCTGCGCGCTCGCTCG	
AAV_-_1	AAV-Seq	CCTCTCTATGGGCAGTCGGTGATGAGGGAGTGGCCAACTCC	
NGG *EMX1* site 1	Targeted PCR	GGGCCTCCTGAGTTTCTCAT	CACCCTAGTCATTGGAGGTGA
NGG *FANCF* site 2	Targeted PCR	CCCAGGTGCTGACGTAGGTA	ATTGACATGCATTTCGACCA
NGA *FANCF* site 1	Targeted PCR	CCAAGGTGAAAGCGGAAGTA	CCCAGGTGCTGACGTAGGTA
NGA *FANCF* site 3	Targeted PCR	CTACCTGCGCCACATCCAT	AAGTTCGCTAATCCCGGAAC
NGG *EMX1* site 1	qPCR	GGGCCTCCTGAGTTTCTCAT	
NGG *FANCF* site 2	qPCR	CCCAGGTGCTGACGTAGGTA	
NGA *FANCF* site 1	qPCR	CCAAGGTGAAAGCGGAAGTA	
NGA *FANCF* site 3	qPCR	CTACCTGCGCCACATCCAT	
Tor1a	qPCR	CTCCCCCTGGAATACAAACA	
APP^{WT}	qPCR	GGTAGGCTTTGTCTTACAGTG	
AAV ITR	qPCR		GGAACCCCTAGTGATGGAGTT
ITR	Southern blot	5' biotin-CACTCCCTCTCTGCGCGCTCG	
U6	Sequencing	GACTATCATATGCTTACCGT	

Supplementary Figure 1.

AAV-seq method (priming sites at the ITR region)

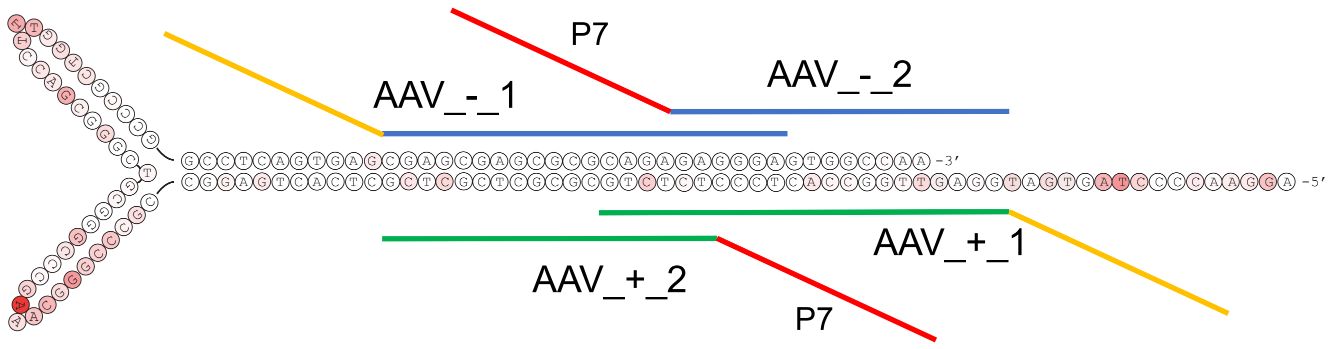

AAV_+_1 GGATCTCGACGCTCTCCCTGGAGTTGGCCACTCCCTCTCTG

AAV_-_1 GGATCTCGACGCTCTCCCTcgagcgagcgcgcagagaggga

AAV_+_2 CCTCTCTATGGGCAGTCGGTGATTCTCTGCGCGCTCGCTCG

AAV_-_2 CCTCTCTATGGGCAGTCGGTGATgagggagtggccaactcc

Supplementary Figure 1. AAVseq method. AAVseq was used to detect AAV vector genome integrations *in vivo*. Nested PCR approach was used with ITR specific primers. Reactions for sense (+) and antisense strand (-) were performed in separate reactions. P7 is Illumina Adaptor sequence.

Supplementary Figure 2.

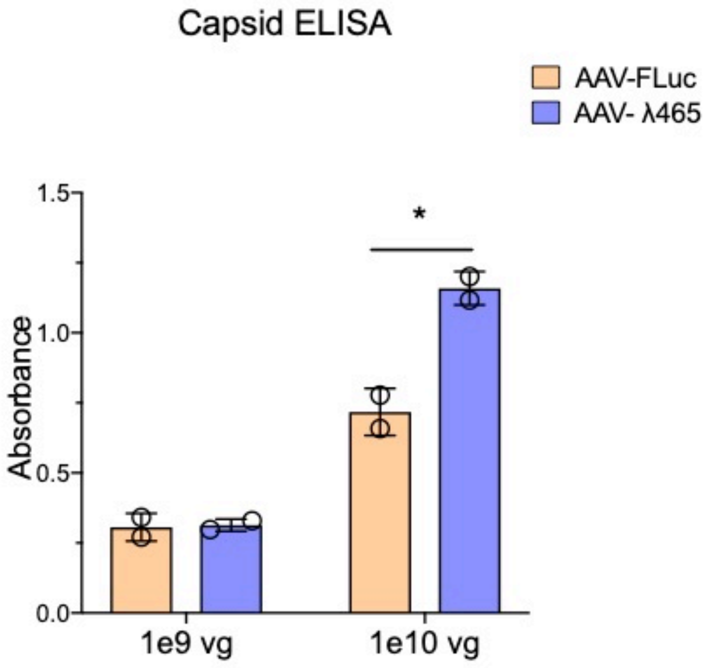

Supplementary Figure 2. AAV capsid ELISA. AAV2-CBA-FLuc-W and AAV2-λ465 was bound to the plate at equal genomic copies (1e9 or 1e10). Vector titers were determined by qPCR against the ITR region. Experiment was performed using two technical replicates. Mean ± SD is shown. (unpaired t-test, p=0.026). Source data are provided as a Source Data file.

Supplementary Figure 3.

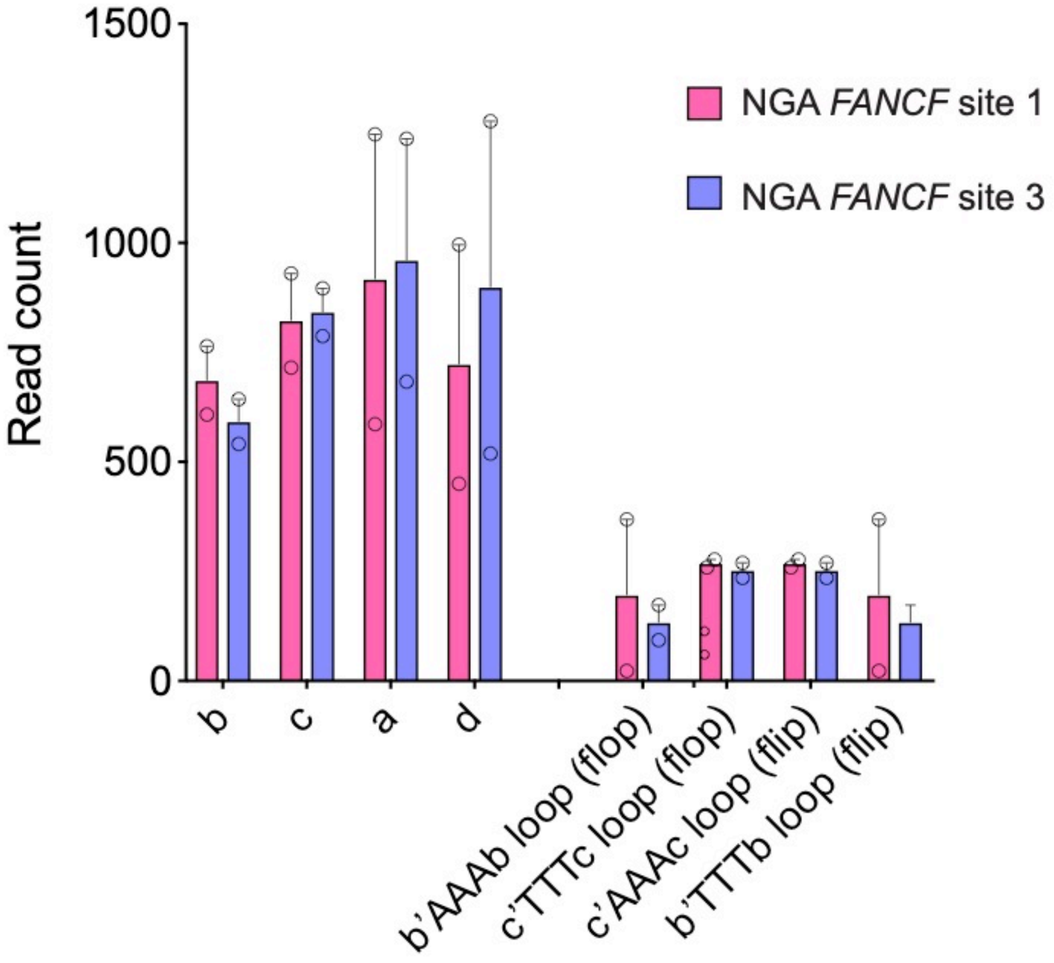

Supplementary Figure 3. **Integrated ITR regions**. Overall read count for different regions of ITR (left) and loop region in flip and flop conformation (right) for two CRISPR target sites (bars represent mean ± SEM). U2-OS cells were transduced with AAV-λ (465 base pairs) and electroporated with Cas9 and gRNA plasmids. Data is from FANCF NGA site 1 and site 3. Source data are provided as a Source Data file.

Type 1 integration: AAV integration with full-length cargo and both ITRs present

clone 1F4: 175/303 nt integration, undetermined (left ITR) and flop (rightITR) configuration

gagCGCGCAGAGA-*a-d'-λ-d-a'-c'-c-b'*-CCCGGGCGTCggg
GCTGCACAACcag aacCAGTGGAGGC
Δ0

clone 4C2: 175/279 nt integration, undetermined (left ITR) and flop (right ITR) configuration

aggGGTTCCTCAG-d-λ-d'-a-c-c'-b-GGCGACCTTTggt
GCTGCACAACcag ggaGGCAAGAGGG
Δ7

Type 2 integration: AAV integration with full-length cargo and one ITR present

clone 2D9: 158/238 nt integration, flop configuration

acgCCCGGGCTTT-*c'-c-a-d'-λ*-GTTGAGTTAGcat
GCCTCCACTGgtt ctgGTTGTGCAGC
Δ0

clone 2F5: 158/230 nt integration, flop configuration

catGCTAACTCAA-*λ-d'-a-c-CGCCCGGGCAaag
CTGCACAACCagt accAGTGGAGGCA
Δ0

clone 5C8: 159/226 nt integration, flop configuration

catGCTAACTCAA-*λ-d'-a-c*-AGGCCGCCCGggc
CTGCACAACCagt accAGTGGAGGCA
Δ0

clone 5E12: 158/238 nt integration, flop configuration

tgcTAACTCAACC-*λ- d'-a-c-c'*-AAGCCCGGGCgtc
CTGCACAACCagt accAGTGGAGGCA
Δ0

Type 3 integration: AAV ITR integration only

clone 3F4: 0/73 nt integration, flip configuration

gggcgACCTTTGGTC-*b'-b-a-d'*-ACTAGGGGTTcct
TGGAGCGGCGgct aacCAGTGGAGGC
Δ10

clone 4A8: 0/50 nt integration, flop configuration

tgaTGGAGTTGGC-*d-a'-b'*-TGAGGCCGGGcga
TGCACAACCAgtg AGTGGAGGCAA
ins1

clone 4A10: 0/61 nt integration, flip configuration

tctAGGAACCCCT-d-a'-c'*-TCACTGAGGCcgc
CTGCACAACCTG CAGTGGAGGCA
ins3

clone 4B11: 0/50 nt integration, flop configuration

tgaTGGAGTTGGC-*d-a'-b'*-TGAGGCCGGGcga
TGCACAACCAgtg AGTGGAGGCAA
ins1

clone 4D5: 0/61 nt integration, flop configuration

tagTGATGGAGTT-*d-a'-b'-GCGACCAAAggt
CTGCACAACCagt accAGTGGAGGCA

Type 4 integration: AAV integration with fragmented cargo and one ITR present

clone 2A5: 53/124 nt integration, flip configuration

cttTGGTCGCCCG-b-a-d'-λ*-GGAGAGATCTagg
GGCGGCTGCAcaa gtgGAGGCAAGAG
Δ9

clone 2B7: 4/70 nt integration, flip configuration

gagATCTGAGGAA-*λ-d-a'-c'*-CACTGAGGCCGCCcgg
GCACAACCAGtgg cagTGGAGGCAAGA
Δ0

clone 2C3 and clone 4A5: 94/165 nt integration, flip configuration

ccggAAAATTTTCA-*λ-d'-a-b-GGGCGACCAaag
ATTCGCACGGctc accAGTGGAGGCAA
Δ24

clone 2C6: 104/176 nt integration, flip configuration

aaacGCCTTAAACC-*λ-d'-a-b-CGGGCGACCAaagg
GCTGCACAACcag aagAGGGCGGCTT
Δ13

clone 3A5 and 3B1: 53/124 nt integration, flip configuration

cttTGGTCGCCCG-b-a-d'-λ*-GGAGAGATCTagg
GGCGGCTGCAcaa gtgGAGGCAAGAG
Δ9

clone 4C11: 94/190 nt integration, flip configuration

tttATGAAAATTT-*λ-d-a'-c'-c-b'-b*-GCGACCAAAGgtc
TGCCTCCACTggt actGGTTGTGCAG
Δ0

clone 4G5: 122/207 nt integration, flop configuration

cgcGAAGAAGAAC-*λ-d'-a-c-c'-b*-CGGGCGTCGggc
CTGCACAACCagt ccaGTGGAGGCA
Δ1

clone 5B6: 96/189 nt integration, flip configuration

ttaaaccGGAAAATTTT-*λ-d'-a-b-b'-c-CGCCCGGGCTttg
CTGCACAACCagt gcaAGAGGGCGGC
Δ7

clone 5F12: 94/165 nt integration, flip configuration

accggAAAATTTTCA-*λ-d'-a-b-CGGGCGACCAaag
ATTCGCACGGctc accAGTGGAGGCA
Δ24

Supplementary Figure 4. Complete list of clones that contain AAV-λ465 vector sequences into NGA *FANCF* site 3. U2-OS cells were treated with AAV-λ465 and were transfected with SpCas9-VRQR and gRNA against NGA *FANCF* site 3. After targeted PCR, column purified PCR fragments were TA cloned and sequenced. After the clone ID, the length of the λ cargo integration/vector integration size is marked. For example, in the case of clone 1F4, 175/303 nt refers to 175bp of the λ cargo integration (no nucleotides missing, full-length), and with the entire integrant length being 303bp (thus the recovered ITR sequence was found to be 128 nt). Integrated ITR configuration was noted either as flip, flop or undetermined, when the length of the ITR was too short to determine the configuration. Coloring of the nucleotides: red: ITR, blue: genomic sequence of the *FANCF* gene, black: λ cargo sequence, green: homologous nucleotides (regions of microhomology) between ITR and genomic cargo. Bold yellow: insertions. Stars (λ* or d*) indicate fragments of the respective regions. Letters (a, b, c, d, a', b', c', d') indicate ITR regions (see Fig. 3h). Capital letters include actual sequences, lowercase nucleotides indicate the hypothetical continuation of the sequence. Solid lines between nucleotides indicate bonds between actual sequences, dotted lines are hypothetical continuations of the sequences. Under the clones, the number of deleted (Δ) and inserted nucleotides (ins) are indicated. Type 2 integrants were defined as having more than 155 nucleotides from the λ cargo (175 nt).
