# Supplementary material for: High levels of AAV vector integration into CRISPR-induced DNA breaks
Source: Nat Commun. 2019 Sep 30;10:4439. doi: 10.1038/s41467-019-12449-2 (PMC6769011; doi:10.1038/s41467-019-12449-2)
Supplement: Supplementary file 21 — Reporting Summary [file 41467_2019_12449_MOESM21_ESM.pdf]

nature research

Corresponding author(s): Casey A. Maguire and Bence Gyorgy

Last updated by author(s): Jul 16, 2019

Reporting Summary

Nature Research wishes to improve the reproducibility of the work that we publish. This form provides structure for consistency and transparency in reporting. For further information on Nature Research policies, see Authors & Referees and the Editorial Policy Checklist.

Statistics

For all statistical analyses, confirm that the following items are present in the figure legend, table legend, main text, or Methods section.

n/a	Confirmed	
☐	☒	The exact sample size (*n*) for each experimental group/condition, given as a discrete number and unit of measurement
☐	☒	A statement on whether measurements were taken from distinct samples or whether the same sample was measured repeatedly
☐	☒	The statistical test(s) used AND whether they are one- or two-sided<br>*Only common tests should be described solely by name; describe more complex techniques in the Methods section.*
☒	☐	A description of all covariates tested
☒	☐	A description of any assumptions or corrections, such as tests of normality and adjustment for multiple comparisons
☐	☒	A full description of the statistical parameters including central tendency (e.g. means) or other basic estimates (e.g. regression coefficient) AND variation (e.g. standard deviation) or associated estimates of uncertainty (e.g. confidence intervals)
☐	☒	For null hypothesis testing, the test statistic (e.g. F, t, r) with confidence intervals, effect sizes, degrees of freedom and P value noted<br>*Give P values as exact values whenever suitable.*
☒	☐	For Bayesian analysis, information on the choice of priors and Markov chain Monte Carlo settings
☒	☐	For hierarchical and complex designs, identification of the appropriate level for tests and full reporting of outcomes
☒	☐	Estimates of effect sizes (e.g. Cohen's d, Pearson's r), indicating how they were calculated

Our web collection on statistics for biologists contains articles on many of the points above.

Software and code

Policy information about availability of computer code

Data collection	No software was used to collect data.
Data analysis	Next-generation sequencing products were analysed using, variously, CRISPResso v2, bwa (version 0.7.17-r1188), and Samtools (v1.7). Virus-Clip (v1.0) was used to analyse whole-genome integrations, which is also reliant on bwa (same version), BLAST alignment tools, and ANNOVAR (version 2018Apr16). Geneious (Java Version 11+28 (64 bit)) was used to plot NGS data. Prism 8.0.2. was used to plot data and perform statistical analysis.

For manuscripts utilizing custom algorithms or software that are central to the research but not yet described in published literature, software must be made available to editors/reviewers. We strongly encourage code deposition in a community repository (e.g. GitHub). See the Nature Research guidelines for submitting code & software for further information.

Data

Policy information about availability of data

All manuscripts must include a data availability statement. This statement should provide the following information, where applicable:

- Accession codes, unique identifiers, or web links for publicly available datasets
- A list of figures that have associated raw data
- A description of any restrictions on data availability

Raw sequencing files have been be uploaded to NCBI's Sequence Read Archive (SRA). A list of uploaded files including SRA IDs are listed in Source Data File. Detailed data analysis is available in the supplementary tables and supplementary data published with this manuscript. The plasmid containing the AAV2-lambda465 is available upon completion of a standard Material Transfer Agreement with The Massachusetts General Hospital. Any other raw data that support the findings of this study are available from the corresponding author. All sequence analysis was run using publicly available programs, which are referred to and referenced in the manuscript along with run parameters where relevant. Quantification of integrant numbers was performed with a script attached to this manuscript as Supplementary Data 18, which also utilized publicly available software.

Field-specific reporting

Please select the one below that is the best fit for your research. If you are not sure, read the appropriate sections before making your selection.

☒ Life sciences ☐ Behavioural & social sciences ☐ Ecological, evolutionary & environmental sciences

For a reference copy of the document with all sections, see nature.com/documents/nr-reporting-summary-flat.pdf

Life sciences study design

All studies must disclose on these points even when the disclosure is negative.

Sample size	No sample size calculation was performed.
Data exclusions	No data was excluded from subsequent data analysis.
Replication	We performed experiments at least on two different occasions on separate biological samples. The detailed number of biological and technical replicates are detailed in each figure legend. We also included a source data file to detail the number of replicates used.
Randomization	Animals were randomly allocated to treatment vs. control groups (with respect to gender).
Blinding	Sample collection was done in a blinded fashion. Genomic DNA analysis and sequencing was not done in a blinded fashion. AAV empty vs full capsid determination was done by a blinded investigator based on transmission electron microscopy experiments.

Reporting for specific materials, systems and methods

We require information from authors about some types of materials, experimental systems and methods used in many studies. Here, indicate whether each material, system or method listed is relevant to your study. If you are not sure if a list item applies to your research, read the appropriate section before selecting a response.

Materials & experimental systems

n/a	Involved in the study	
☐	☒	Antibodies
☐	☒	Eukaryotic cell lines
☒	☐	Palaeontology
☐	☒	Animals and other organisms
☒	☐	Human research participants
☒	☐	Clinical data

Methods

n/a	Involved in the study	
☒	☐	ChIP-seq
☒	☐	Flow cytometry
☒	☐	MRI-based neuroimaging

Antibodies

Antibodies used	anti-AAV2 antibody A20, American Research Products catalog number 03-61055,<br>; anti-AAV2, clone A20, Biotinylated, American Research Products, catalog number 03-61555
Validation	Citations for A20 cat# 03-61055: citations: https://www.citeab.com/antibodies/5710671-03-61055-anti-adeno-associated-virus-aav2-intact?utm_campaign=Widget+All+Citations&utm_medium=Widget&utm_source=American+Research+Products&utm_term=American+Research+Products<br>We validated antibodies by using a positive (AAV2 particles) and negative control (vehicle, PBS) in all experiments.

Eukaryotic cell lines

Policy information about cell lines

Cell line source(s)	293T, source ATCC; U2-OS cells Homo sapiens bone osteosarcoma, ATCC® HTB-96™
Authentication	None of the cell lines were authenticated
Mycoplasma contamination	All cell lines tested negative for mycoplasma contamination.
Commonly misidentified lines (See ICLAC register)	Not applicable

Animals and other organisms

Policy information about studies involving animals; ARRIVE guidelines recommended for reporting animal research

Laboratory animals	C57BL/6 mice were used in this study. Animals were purchased from Charles River Laboratories. The details of the animals with respect to age and gender are included in Source Data file.
Wild animals	The study did not use wild animals.
Field-collected samples	Not applicable.
Ethics oversight	All experiments were performed in compliance with ethical regulations approved by the Animal Care Committee of Massachusetts General Hospital.

Note that full information on the approval of the study protocol must also be provided in the manuscript.
